# Patterns and predictors of cancer‐specific patient health portal usage among patients with cancer: results from the UWCCC Survivorship Program

**DOI:** 10.1002/cam4.4234

**Published:** 2021-08-28

**Authors:** Rebecca P. Luoh, Amye J. Tevaarwerk, Thevaa Chandereng, Elena M. Smith, Cibele B. Carroll, Hamid Emamekhoo, Mary E. Sesto

**Affiliations:** ^1^ School of Medicine and Public Health University of Wisconsin Madison Wisconsin USA; ^2^ University of Wisconsin Carbone Cancer Center Madison Wisconsin USA; ^3^ UW Health Madison Wisconsin USA; ^4^ Present address: Dept of Biostatistics Columbia University New York NY 10032 USA

**Keywords:** cancer, electronic health records, health records, personal, patient portals

## Abstract

**Background:**

Portals can assist patients in managing their healthcare. Understanding how patients with cancer use portals can facilitate improvements in patient engagement in cancer care. This study sought to determine if patients with cancer used portals differently for cancer versus noncancer purposes. The effects of geographic residence (rural vs. urban residence) and cancer stage on portal usage were also investigated.

**Methods:**

We conducted a retrospective analysis of portal usage by patients seen at an NCI‐designated cancer center between 2015 and 2019. Demographics, cancer characteristics, and portal usage (number of successful logins, messages sent, and results viewed) were extracted. Messages sent and results viewed in the portal were deemed oncologist‐specific and cancer specific if sent to or ordered in medical oncology departments, respectively.

**Results:**

The analysis included a total of 5950 patients with cancer. Patients were *less* likely to send and view oncologist‐specific messages compared to non‐oncologist‐specific messages. They were also *less* likely to view cancer results compared to noncancer results. Compared to urban counterparts, patients residing in rural areas had lower odds of having any logins and logged in less frequently during the year of diagnosis. Compared to patients with non‐metastatic disease, individuals with metastatic disease were more likely to become frequent portal users.

**Conclusions:**

Patients may use portals differently for cancer versus noncancer purposes; urban residence and metastatic cancer were associated with more frequent usage. Further investigation can inform interventions to increase accessibility for groups at a disadvantage related to the use of this technology and to help patients better leverage portals to manage their cancer.

## Graphical abstract

Healthcare systems have come to increasingly rely on patient health portals to engage patients; however, patients with cancer may use portals for cancer purposes differently than for noncancer purposes. Our analysis of nearly 6000 patients with cancer suggests that there is a difference in cancer versus noncancer use and that factors such as age, rural residence, and non‐metastatic stage make patients less likely to access portals.

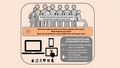

## 1 INTRODUCTION

Nearly 40% of the U.S. population will be diagnosed with cancer during their lifetime; managing the complexity of cancer and subsequent treatment can place a substantial burden on patients and/or caregivers.^1^ Patients may have multiple providers from different specialties, which increases the risk for care fragmentation.^2^, ^3^, ^4^ Patients and caregivers may struggle with coordinating appointments, managing medications and side‐effects, coping with financial challenges, and communicating across healthcare systems. Patients with cancer are often challenged by such complex self‐management tasks and may lack tools or resources for addressing this cancer care‐related work.^3^, ^5^, ^6^, ^7^, ^8^, ^9^

The patient health portal (“portal”) is an information technology tool that may help patients with cancer manage cancer care‐related work. A portal integrates with, or is tethered to, a healthcare system’s electronic health record (EHR) to support patients with managing their health.^10^, ^11^ Portals may provide patients access to their health information, such as doctors’ notes and lab results.^12^ They also give patients the ability to message their healthcare providers,^12^ which may help coordinate care, including between members of their multidisciplinary team.^13^, ^14^, ^15^ When patients with cancer have access to portals, they feel more informed and more in control of their disease^13^, ^14^, ^15^ as well as participating more fully during in‐person consultations.^13^

Patients may use portals differently to manage cancer versus noncancer care‐related work—Johnson et al found that patients with cancer were *more* likely to use portals when compared to patients without cancer.^16^ But, a portal provided by a healthcare system can be used for multiple provider encounters including cancer and noncancer care, such as when a patient with cancer messages primary care (noncancer‐related use) and also checks on cancer labs ordered by the oncologist (cancer‐related use). To date, published literature has examined *generalized* portal usage by patients with cancer, rather than whether the portal was used to address cancer care‐related work.^17^, ^18^, ^19^, ^20^ Differentiation between portal usage for cancer versus noncancer could influence strategies that help patients use the portal for cancer‐related purposes. For example, implementation of an online symptom questionnaire is a potential strategy for managing symptoms related to cancer treatment.^21^

In addition to understanding how patients with cancer might use portals to manage cancer care‐related work, it is important to understand which populations may struggle to use portals for this work. Patients who are older, African American, Hispanic, or Spanish‐speaking are less likely to use portals.^17^, ^18^, ^19^, ^22^ Rurality may also affect usage by patients with cancer, given widespread issues with broadband and cellular data access in rural areas of the United States.^23^, ^24^ Recent NCI‐funded research describes a digital rural‐urban health IT divide,^25^ and Arcury et al found that patients receiving care at rural clinics used portals less than those at urban clinics.^24^ However, research addressing whether rurality affects portal usage among patients with cancer remains limited. Additionally, the cancer stage usually drives treatment complexity (e.g., DCIS is generally less complex than Stage III or IV breast cancer) and whether the stage affects portal usage has not been explored.

Our objective was to determine patterns and predictors of cancer‐specific portal use among patients with cancer. Thus, we collected and analyzed patient demographic, cancer, and portal usage information at a single National Cancer Institute‐designated comprehensive cancer center.

## 2 METHODS

### 2.1 Setting

This study was conducted at the University of Wisconsin Carbone Cancer Center (UWCCC). UWCCC is a part of UW Health, an integrated health system of the University of Wisconsin‐Madison and has a primary clinical catchment area including multiple rural Wisconsin counties.^26^ The EHR vendor is Epic® (Epic Systems; Verona, WI, USA) and Epic’s MyChart® is the secure, online patient health portal that patients use to access health information within UW Health’s EHR. MyChart® has been available to patients at UWCCC since 2010. This study was exempted from the University of Wisconsin Institutional Review Board (IRB) review.

### 2.2 Analysis population

Patients included in the analysis met the following criteria: (1) age ≥18; (2) ≥2 visits to UWCCC medical oncology departments; (3) diagnosed with cancer between 1/1/2015 and 12/31/2019; and (4) met criteria for inclusion on UWCCC’s EHR‐based Cancer Registry. The UWCCC EHR‐based registry relies on Epic’s Healthy Planet® functionality and provides real‐time data on any patients with an active cancer diagnosis listed on the EHR problem list. Patients were included whether alive or deceased. Patients were excluded if they had: (1) only non‐solid tumor diagnoses, (2) only had a single UWCCC visit as this was likely consultative, (3) incomplete patient status (e.g., no data about alive or deceased) or (4) incomplete MyChart account status (e.g., no data about the account being declined, pending activation, activated or inactivated).

### 2.3 Variables, source, and extraction

For patients meeting the above criteria, we extracted demographics and cancer characteristics as presented in Figure S1. Portal account status and usage were extracted from Epic Clarity® Tables using a structured query language and matched to patient data using medical record numbers. We sought to explore patients’ account status but chose not to rely solely on MyChart account status because this data reflects the last account value (e.g., declined, pending activation, activated or inactivated) and may obscure prior use (e.g., deceased patient accounts are automatically deactivated, but data about account usage remains). Thus, we separated patients into “no usage” versus “any usage” if they had 0 logins or ≥1 login during the study timeframe, respectively. Among patients who had any usage (≥1 login), to categorize patients by frequency of portal use, we divided patients with accounts into quartiles based on the number of successful logins, similar to Tsai et al,^27^ as there is no agreed upon the definition of a “high frequency user.” Patients with ≥75th percentile of logins were deemed “frequent portal users” while those with ≤25th percentile of logins were deemed “infrequent portal users.”

### 2.4 Portal usage

Portal usage data were extracted by counts (e.g., number of successful logins, number of messages sent, *etc*.) over two distinct timeframes.

#### 2.4.1 Year‐of‐diagnosis

The year following a cancer diagnosis is generally the timeframe in which appointments and testing are concentrated, requiring frequent care‐related work.^3^ Data were extracted between the date of patient’s cancer diagnosis and one year after, within January 1st, 2015 to June 29th, 2020—latter is the date of the data pull. For those who did not have an account and log in during the entire year (e.g., they signed up for an account and/or died during that timeframe), data were standardized and presented as counts per year (hereafter known as “year‐of‐diagnosis”). For example, a patient was diagnosed on Jan 1, 2018, passed away on 6/30/2018, and logged in to MyChart 5 times between diagnosis and death. The timeframe of MyChart use is thus 0.5 years, and number of logins per year during year‐of‐diagnosis is 5/0.5 = 10.

#### 2.4.2 Years‐of‐activity

Following the intense care‐related work during diagnosis and active treatment, cancer survivors usually experience a period of follow‐up, surveillance, and adaption to functional limitations secondary to cancer and its treatments.^3^ For this timeframe, data were extracted between January 1st, 2015 and December 31st, 2019. As some patients did not have an active account during the entire period described, data were standardized as above, and presented as counts per year (hereafter known as “years‐of‐activity”).

#### 2.4.3 Cancer‐specific usage

The team examined patient portal activities that might capture cancer‐specific usage. This included appointments requests, messages (logged in Epic as “Medical Advice Requests”), and reviews of any results such as labs or imaging in the portal (logged in Epic as “Test Results”). Appointment requests could not be used because UW Health has not enabled appointment scheduling for oncology via the portal. However, messages and results could be refined to examine cancer‐specific portal usage as follows.

#### 2.4.4 Oncologist‐specific messages

Patient‐initiated messages routed to medical oncology departments were considered oncologist‐specific messages, to manage cancer care. Non‐oncologist‐specific messages, assumed to be used to manage general care, were calculated by subtracting the number of oncologist‐specific messages from the total number of patient‐initiated messages. The number of messages, between 2015 and 2019, were pulled and standardized to counts per year.

#### 2.4.5 Oncologist‐ordered results

Orders for tests placed within medical oncology departments were considered to be ordered by an oncologist (“cancer results”). Orders placed outside of the medical oncology departments were considered not associated with cancer diagnosis (“noncancer results”). The orders results described in this research are related to pathology, laboratory (including tumor markers), and radiology (including staging scans). The percentage of cancer results viewed was calculated after extracting the number of cancer results viewed and dividing by the number of cancer results released to the patient portal. The percentage of noncancer results viewed was calculated similarly. For this part of the analysis, we included only patients who had ≥1 cancer result and ≥1 noncancer result that was viewable on the portal. This allowed us to compare the rate of cancer and noncancer results viewing for each patient.

### 2.5 Statistical analysis

Data analysis was conducted with the R statistical software version R 4.0.1. (R Foundation for Statistical Computing, Vienna, Austria). Means, standard deviations, and proportions were calculated for continuous and categorical variables. For portal usage, we assessed the relationship between the likelihood of any usage with ≥1 login between January 1st, 2015 and December 31st, 2019 and age, sex, race, residence area, insurance, and stage, and the two‐way interactions between these variables using a logistic regression model. We assessed the relationship between the number of logins within years‐of‐activity and year‐from‐diagnosis with the same variables, as well the two‐way interactions between them, using a Poisson regression model. We used a paired Wilcoxon rank‐sum test to compare the number of logins during those two timeframes. We used a stratified Wilcoxon rank‐sum test to compare mobile and web logins for urban (Rural‐Urban Continuum Codes (RUCC) ≤ 3) or rural (RUCC ≥ 4) areas. Chi‐square test and one‐way ANOVA were used to assess risk factors associated with frequent and infrequent users as presented in Table 1. Yates’s correction was not applied for the chi‐square test due to the overly conservative nature of the correction.^28^ A q‐value (*P_adj_*) corrected for false discovery rate (FDR) of 0.05 was considered statistically significant. For cancer versus noncancer usage, we used paired Wilcoxon rank‐sum test to compare the number of oncologist‐specific and non‐oncologist‐specific messages sent and percentage of results viewed for each patient.

**TABLE 1:** Demographics and cancer characteristics of patients based on portal accounts status and usage

Variables	All patients n = 5950 (100.0%)	No usage n = 2052 (34.5%)	Infrequent users^a^ n = 973 (16.4%)	Regular users^a^ n = 1951 (32.7%)	Frequent users^a^ n = 974 (16.4%)
**Age**, mean (IQR)	64 (17)	67 (17)	65 (18)	63 (12)	60 (19)
**Sex**, n (%)					
Female	3185 (53.5%)	948 (46.2%)	551 (56.6%)	1,134 (58%)	552 (56.7%)
Male	2765 (46.5%)	1104 (53.8%)	422 (43.4%)	817 (42%)	422 (43.3%)
**Race**, n (%)					
White	5587 (93.9%)	1869 (91.1%)	912 (93.7%)	1868 (96%)	938 (96.3%)
Non‐White^b^	318 (5.3%)	167 (8.1%)	56 (5.8%)	68 (3.5%)	27 (2.8%)
Unavailable	45 (0.8%)	16 (0.8%)	5 (0.5%)	15 (0.8%)	9 (0.9%)
**Ethnicity**, n (%)					
Not Hispanic	5815 (97.8%)	1993 (97.1%)	957 (98.4%)	1916 (98.2%)	949 (97.4%)
Hispanic	110 (1.8%)	50 (2.5%)	12 (1.2%)	27 (1.4%)	21 (2.2%)
Unavailable	25 (0.4%)	9 (0.4%)	4 (0.4%)	8 (0.4%)	4 (0.4%)
**Rural/Urban** ^c^, n (%)					
Urban	4271 (71.8%)	1284 (62.6%)	732 (75.2%)	1522 (79%)	733 (75.3%)
Rural	1508 (25.3%)	656 (31.9%)	218 (22.4%)	400 (21%)	234 (24.0%)
Unavailable	171 (2.9%)	112 (5.5%)	23 (2.4%)	29	7 (0.7%)
**Insurance**, n (%)					
Medicare/Medicaid	2595 (43.7%)	989 (48.1%)	417 (42.9%)	835 (48%)	354 (36.3%)
Private	2326 (39.1%)	543 (26.5%)	397 (40.8%)	890 (51%)	496 (50.9%)
Other	80 (1.3%)	63 (3.1%)	4 (0.4%)	4 (0.2%)	9 (0.9%)
Unknown	949 (15.9%)	457 (22.3%)	155 (15.9%)	222	115 (11.8%)
**Cancer Type**, n (%)					
Breast	1286 (21.6%)	270 (13.2%)	250 (25.7%)	546 (28%)	220 (22.6%)
Lung	784 (13.1%)	394 (19.2%)	94 (9.7%)	193 (10%)	103 (10.6%)
Colon and Rectum	432 (7.3%)	144 (7.0%)	70 (7.2%)	144 (7.3%)	74 (7.6%)
Prostate	286 (4.8%)	104 (5.1%)	57 (5.9%)	89 (4.6%)	36 (3.7%)
Other	2895 (48.7%)	1027 (50.0%)	462 (47.5%)	912 (46.7%)	494 (50.7%)
Unknown	267 (4.5%)	113 (5.5%)	40 (4.1%)	67 (3.4%)	47 (4.8%)
**Cancer Stage** ^d^, n (%)					
Non‐Metastatic	1620 (27.2%)	406 (19.8%)	261 (26.8%)	655 (34%)	298 (30.6%)
Metastatic	1256 (21.1%)	355 (17.3%)	163 (16.8%)	424 (22%)	314 (32.2%)
Unknown	3074 (51.7%)	1291 (62.9%)	549 (56.4%)	872 (45%)	362 (37.2%)

IQR = Interquartile range, No usage = Patients with 0 logins in the study timeframe.

^a^ Patients with ≥75th percentile of logins were deemed “frequent users”, those with logins between 25th and 75th percentile “regular users”, while those with ≤25th percentile of logins were deemed “infrequent users.

^b^ Non‐White is the collapsed category include black, multiracial, and others.

^c^ Defined using Rural‐Urban Continuum Codes (RUCC). Urban = RUCC 1–3 and rural = RUCC 4–9.

^d^ Stages 0–III are considered Non‐Metastatic, and stage IV is considered metastatic regardless of cancer type. Since March 2019, stage data have been required to close an encounter at the UWCCC. Unknown = where the stage was not recorded discretely in the Electronic Health Record.

## 3 RESULTS

### 3.1 Demographics and cancer characteristics

We identified 5959 patients who met the inclusion criteria; 9 patients with incomplete data were excluded. Thus, a total of 5950 patients were included in the final analysis; 1946 (32.7%) were deceased. Table 1 presents the study population’s demographics and cancer characteristics.

### 3.2 Factors associated with any usage

As shown in Table 2, factors associated with any portal usage were sex (male OR: 0.72; 95% CI (0.63, 0.82)), race (white OR: 2.46; 95% CI (1.85, 3.26)), insurance coverage (private OR: 8.94; 95% CI (3.78, 21.21)), and place of residence (rural OR: 0.50; 95% CI (0.43, 0.58)). Cancer site, and cancer stage were not associated. However, there is an interaction effect between age and private insurance, for a person with private insurance, as age increases, they are less likely to have any portal usage (OR: 0.97; 95% CI (0.96, 0.99)).

**TABLE 2:** Predictors of the portal account status and usage by patients with cancer

Variables	Any Usage^a^ OR (95% CI)^c^	Years‐of‐activity^b^ RR (95% CI)^d^	Year‐of‐diagnosis^b^ RR (95% CI)^d^
**Age**	0.99 (0.98–1.00)	0.991 (0.990–0.991)	0.991 (0.991–0.992)
**Sex**			
Female	Reference	Reference	Reference
Male	0.71 (0.62–0.81)	1.01 (1.00–1.01)	1.03 (1.02–1.04)
**Race**			
Non‐White	Reference	Reference	Reference
White	2.45 (1.84–3.26)	1.67 (1.63–1.72)	1.58 (1.55–1.61)
**Rural/Urban residence**			
Urban	Reference	Reference	Reference
Rural	0.50 (0.43–0.57)	0.86 (0.73–0.99)	0.53 (0.47–0.61)
**Insurance Status**			
Medicare/Medicaid	Reference	Reference	Reference
Private	8.93 (3.78–21.20)	2.37 (2.24–2.51)	3.13 (3.00–3.27)
**Stage**			
Metastatic	Reference	Reference	Reference
Non‐Metastatic	0.97 (0.79–1.17)	0.82 (0.81–0.83)	0.83 (0.82–0.83)
**Interactions between variables** (*X* indicates the variables being tested for interaction)			
“Age” *X* “rural residence”	*NA*	0.987 (0.986–0.988)	0.990 (0.990–0.991)
“Age” *X* “private insurance”	0.97 (0.95–0.98)	0.987 (0.986–0.988)	0.983 (0.983–0.984)
“White race” *X* “rural residence”	*NA*	2.34 (2.04–2.68)	2.99 (2.67–3.37)
“Rural residence” *X* “private insurance”	*NA*	0.92 (0.89–0.94)	0.84 (0.82–0.85)

Non‐White is the collapsed category include black, multiracial, and others. NA = Non‐applicable.

^a^ Logistic regression using whether patients had at least one login during the study timeframe.

^b^ Poisson regression using counts of logins.

^c^ OR: Odds Ratio, CI: Confidence Interval.

^d^ RR: Rate Ratio.

### 3.3 Factors associated with frequency of usage

Among patients with at least one account login, frequent users logged in a median of 109 times per year (range 68–821, IQR 82) compared to infrequent users at a median of 4 times per year (range 0–11, IQR 6). The most common portal functionalities used by patients who logged in at least once during both timeframes (years‐of‐activity and year‐of‐diagnosis) are shown in Table 3. Patients logged in more during the year‐of‐diagnosis than during years‐of‐activity (difference of medians = 2.99). Although there was significantly more variation in the number of logins during year‐of‐diagnosis with higher ranks, the median of logins of years‐of‐activity was higher. While more patients had no logins during year‐of‐diagnosis compared to during years‐of‐activity (43.7% vs. 34.5%), there was also a group of patients that logged in much more frequently (up to multiple times daily during year‐of‐diagnosis) as shown in Figure S2.

**TABLE 3:** Most common clinically meaningful portal activity and functionality used by patients with cancer with at least one login^a^

MyChart functionality	Usage in counts per year^b^, median (min, max; IQR)	
	Years‐of‐activity	Year‐of‐diagnosis
Logged in	31 (0, 821; 56)	51 (0, 2274; 119)
Viewed past and upcoming visits	25 (0, 778; 47)	38 (0, 1913; 107)
Viewed Health Maintenance	16 (0, 771; 36)	17 (0, 1179; 74)
Viewed test results	15 (0, 721; 27)	23 (0, 1029; 61)
Viewed and acted on messages	12 (0, 514; 23)	19 (0, 1217; 49)
Viewed hospital admission summary	8 (0, 643; 18)	7 (0, 1219; 38)
Viewed Medical History	6 (0, 58; 14)	6 (0, 1066; 31)

IQR = Interquartile range

^a^ Functionalities “Logged out” and “Provider List Widget” (logged in Epic when patient lands on the homepage) removed for clinical irrelevance.

^b^ Usage is normalized to counts per year by adjusting for when patients signed up for MyChart and when patients died (if applicable) between 1/1/2015 and 12/31/2019 for years‐of‐activity, and between patients’ cancer diagnosis date and one year after for year‐of‐diagnosis.

Factors associated with portal usage during year‐of‐diagnosis and years‐of‐activity were age, sex, race, and insurance coverage, as younger, male, white, and privately insured patients were more likely to use the portal (Table 2). Metastatic disease was also increased the likelihood of portal usage during both timeframes. The cancer site was not associated with account utilization. A significant interaction between the variables age, rurality, and insurance were found. Whether patients lived in rural or urban areas was associated with log in during year‐of‐diagnosis (Rate Ratio (RR): 0.54; 95% CI (0.47, 0.61)), although not during years‐of‐activity ((RR): 0.86; 95% CI (0.73, 1.00)). Regardless of their geographic residence, patients logged into the portal through the web platform more often compared to the mobile platform (mean difference in proportions (web – mobile): 0.63; 95% CI (0.58, 0.67)).

Patients sent fewer oncologist‐specific messages compared to non‐oncologist‐specific messages during years‐of‐activity and year‐of‐diagnosis, but overall, the use of messaging was low. During year‐of‐diagnosis, patients sent a median of 0 oncologist‐specific (range 0–371, IQR 9) and 2 non‐oncologist‐specific messages (range 0–526, IQR 13) per year. During years‐of‐activity, patients sent median of 0.3 oncologist‐specific (range 0–271, IQR 3) and 1.2 non‐oncologist‐specific messages (range 0–269, IQR 7) per year. Similarly, patients viewed results for cancer care purposes less frequently compared to noncancer during year‐of‐diagnosis and years‐of‐activity. See also Figure S3.

## 4 DISCUSSION

Understanding how patients with cancer use portals to access their personal medical records and message their cancer care team is vital. Cancer diagnoses and treatment are complicated, and patients and caregivers must often assist in managing their cancer care.^3^, ^5^, ^7^, ^8^, ^9^ Portals are a tool that may help patients with chronic diseases such as cancer undertake such care‐related work.^13^, ^14^, ^15^ This analysis adds to the literature in exploring how patients with cancer utilize portals by assessing portal usage at one NCI‐designated comprehensive cancer center serving a substantial rural population. Rural patients with cancer represented around 25% of all individuals with solid tumors with visits to UWCCC medical oncology departments between January 1st, 2015 and December 31st, 2019. Moreover, to our knowledge, this is the first study to examine how patients with cancer use portals *specifically* with respect to cancer. Thus, our study reveals not only how patients with cancer use portals in general, but how they use portals *in relation to cancer*.

At our institution, patients use portals differently for their cancer compared to noncancer purposes. Patients appeared slightly less likely to use oncologist‐specific messaging, although the overall volume of *any* messages was low. As a system, UW Health does promote the use of portal messaging, and UWCCC oncology departments typically review appropriate use for oncology needs. To address acute needs, the UWCCC has an oncology triage line staffed by oncology nurses during business hours that offers a timely answer to the patients’ needs. Similarly, patients were also less likely to view cancer results in comparison with noncancer results, perhaps because at the UWCCC, cancer testing often occurs in proximity with provider visits for review of these results. Our results also suggest that patients do not have much change in behavior related to portal usage once starting their cancer treatment. Future studies into how patients use portals for cancer specifically can help develop strategies to support patients in fully leveraging a portal’s potential. For example, personalized messages and monitoring physical activity can help with the adoption of a healthier lifestyle over the cancer care continuum.^29^ Our study also investigated how patient demographic and cancer characteristics correlate with portal usage. Similar to Gerber et al., we found that younger, white patients with cancer are more likely to log in.^17^ Insurance type was not significant in a noncancer population,^27^ but in our study, patients covered by private insurance were more likely to log in. It is important to note, however, that non‐private insurance (e.g., Medicaid/Medicare) may have had significant interaction with age in the case of Medicare and resource access in the case of Medicaid. Thus, the effect of insurance on logins might be driven by those factors, since portal access is not typically determined by insurance status in U.S. health systems. We found that patients living in rural areas are less likely to have any portal usage and are also log in less frequently during year‐of‐diagnosis. In other words, rurality was associated with both lower likelihood and lower frequency of portal use. While our study cannot speak to the causality of this association, we note that rural parts of our state have more limited access to both broadband and cellular services.^30^ Our study also adds uniquely to the literature with regards to the cancer stage. A metastatic diagnosis did not predict for ever having used a portal but was associated with frequency of usage. This suggests that patients with metastatic cancer use portals more frequently *if* they have an account. More work is needed to further investigate this finding. We hypothesize that patients with metastatic cancer may experience more symptoms with disease progression that necessitate frequent visits with providers and changes to therapeutics. While patients with metastatic cancer may have lower functional status due to their higher disease burden, this can depend on cancer—for example, patients with metastatic breast cancer may do well for years. Additionally, we are aware through anecdotal reports that caregivers communicate with providers directly through the patients’ MyChart account, as opposed to through proxy accounts. Thus, patients’ MyChart usage may also reflect caregiver usage for very ill patients.

Viewing results was the second most used functionality (excluding logging in) in the year after diagnosis and third during the other years of follow‐up. Other frequently used portal functionalities in our population (viewing messages and visits) have been reported as common functionalities in previous studies of patients with cancer.^17^ One intriguing finding is that as patients’ portal usage decreased, the proportion of patients with an unknown cancer stage increased: 37.2% among frequent users, 56.4% among infrequent users, and 62.9% among patients with no account. We hypothesize that providers who were less likely to discretely document stage (and thus, possibly less comfortable with EHRs in general) may be less likely to provide patients with opportunities to sign up for or use a portal. The lack of providers’ support for patient engagement with the portal account has been previously reported in the literature as a barrier to patient portal utilization.^31^, ^32^ Of note, almost half of our patients (51.7%) did not have stage documented, which may have affected the overall power for comparison between stages.

Weaknesses include a patient population that is predominantly white (93.9%) and non‐Hispanic (97.7%), making our results less generalizable to other populations. For cancer usage (messages and results), because we limited the inclusion criteria to medical oncology departments, our analysis does not include portal activity for surgical or radiation oncology purposes. Thus, we may *underestimate* the true extent to which patients with cancer use a portal to manage their cancer care. For noncancer use, we did not include the count of comorbidities in the analysis. As the data were extracted from EHR utilizing structured query language, we are conscious of the accuracy of noncancer comorbidity description in the problem list. Previous reports pointed to the discrepancy between the number of comorbidities when comparing problem lists and free‐text notes, as the latter tend to be more precise.^33^ Moreover, the influence of comorbidities can be confounded with age, as it tends to increase with aging, and among patients with cancer it is difficult to precisely account for the impact of time since diagnosis, cancer treatments rendered, and expected outcome from cancer.

We plan to continue our examination into cancer versus noncancer portal usage, by investigating whether there are patients who have high cancer usage but low noncancer usage and vice versa and patterns of use among frequent portal users. Further research into how patients with metastatic cancer use portals would also be helpful to better support this patient population, who are often under high physical and emotional duress. Ultimately, a better understanding of how patients with cancer use portals will improve the ability to design and create strategies to extend the potential benefits to populations underserved by this technology.

## 5 CONFLICTS/DISCLOSURES

Amye J. Tevaarwerk – Epic Systems (family member)

Cibele B. Carroll ‐ None

Elena M. Smith ‐ None

Hamid Emamekhoo ‐ None

Mary E. Sesto ‐ None

Rebecca P. Luoh ‐ None

Thevaa Chandereng – None

## AUTHOR CONTRIBUTIONS

Amye J. Tevaarwerk: conceptualization, data curation, formal analysis, funding acquisition, investigation, methodology, project administration, resources, supervision, validation, visualization, writing ‐ original draft, and writing ‐ review and editing.

Cibele B. Carroll: formal analysis, investigation, methodology, project administration, supervision, validation, visualization, writing ‐ original draft, and writing ‐ review and editing.

Elena M. Smith: data curation, formal analysis, supervision, validation, visualization, writing ‐ original draft, and writing ‐ review and editing.

Hamid Emamekhoo: conceptualization, formal analysis, investigation, methodology, project administration, resources, supervision, validation, visualization, writing ‐ original draft, and writing ‐ review and editing.

Mary E. Sesto: conceptualization, data curation, formal analysis, funding acquisition, investigation, methodology, project administration, resources, supervision, validation, visualization, writing ‐ original draft, and writing ‐ review and editing.

Rebecca P. Luoh: conceptualization, data curation, formal analysis, software, investigation, methodology, project administration, supervision, validation, visualization, writing ‐ original draft, and writing ‐ review and editing.

Thevaa Chandereng: data curation, formal analysis, software, investigation, methodology, project administration, supervision, validation, visualization, writing ‐ original draft, and writing ‐ review and editing.

## CONFLICT OF INTEREST STATEMENT

The authors confirm that there are no conflicts of interest.

## ETHICAL APPROVAL STATEMENT

This study was exempted from review by the University of Wisconsin IRB.

## Supporting information

Supplementary Material

## Data Availability

Data were extracted from the UW Health Healthy Planet® Cancer Registry, an Electronic Health Records ‐ based cancer registry. Portal account usage data were extracted from Epic Clarity® Tables. Data elements will be available upon direct request to the corresponding author (AJT) to ensure that we protect the subject’s privacy.
