# Supplementary material for: Patterns and predictors of cancer‐specific patient health portal usage among patients with cancer: results from the UWCCC Survivorship Program
Source: Cancer Med. 2021 Aug 28;10(20):7373–82. doi: 10.1002/cam4.4234 (PMC8525111; doi:10.1002/cam4.4234)
Supplement: Supplementary file 1 — Supplementary Material [file CAM4-10-7373-s001.pdf]

Figure 1S. Study variables and analysis categories.

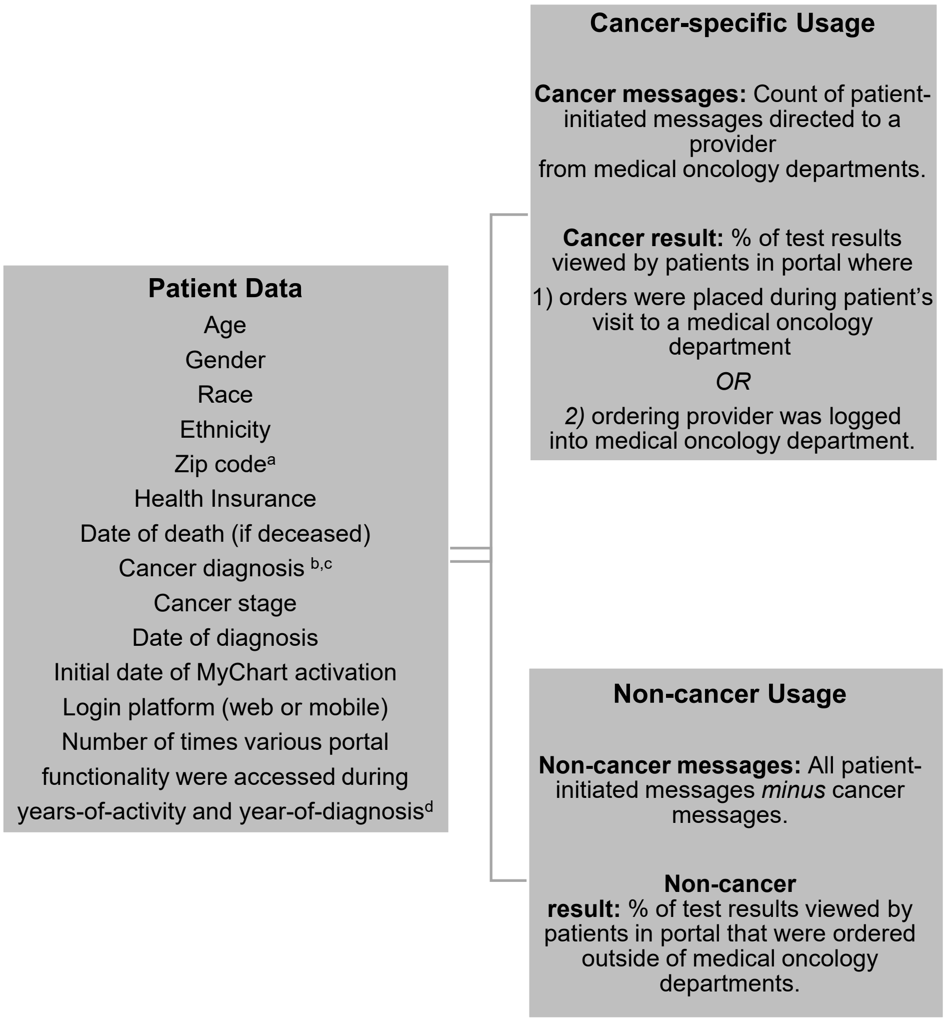

[a]Used to derive Rural-Urban Continuum Codes (RUCC)[26]

[b]The most recent discrete Diagnosis and Stage data were extracted. Since 2019, UWCCC close visit validation logic requires medical oncology providers to add a malignant diagnosis to the Problem List and stage patients discretely.

[c]Cancer diagnosis were grouped using Epic's GROUPER_CANCERTYPES Clarity table, which is based on American Joint Committee on Cancer's site categories.

[d]Year-of-diagnosis is the timeframe between when patients was diagnosed with cancer and one year after, also taking when patients signed up for account and death date (if applicable) into account. Years-of-activity is the timeframe between when patients signed up for account and death date (if applicable) within 1/1/2015 and 12/31/2019.

Figure 2S. Box Plot with the ranking of number of logins per year.

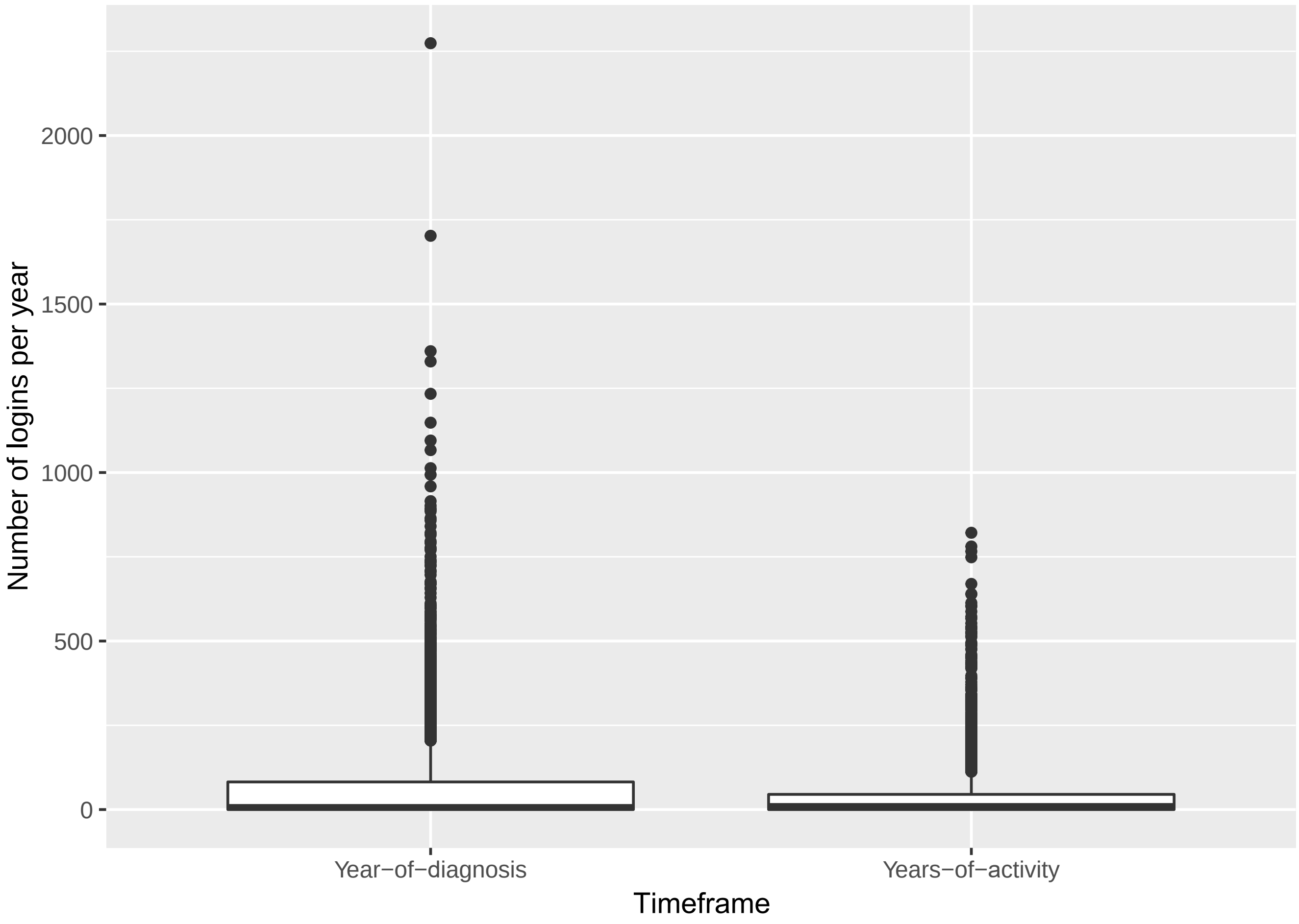

Number of logins were normalized by the respective timeframe and presented as number of logins per year. Year-of-diagnosis is the timeframe between when patients were diagnosed with cancer and one year after, also taking when patients signed up for account and death date (if applicable) into account. Years-of-activity is the timeframe between when patients signed up for the account and death date (if applicable) within 1/1/2015 and 12/31/2019.

Figure 3S. Histogram with the proportion of results viewed by patient in MyChart comparing cancer vs noncancer related results.

A. Year-of-diagnosis

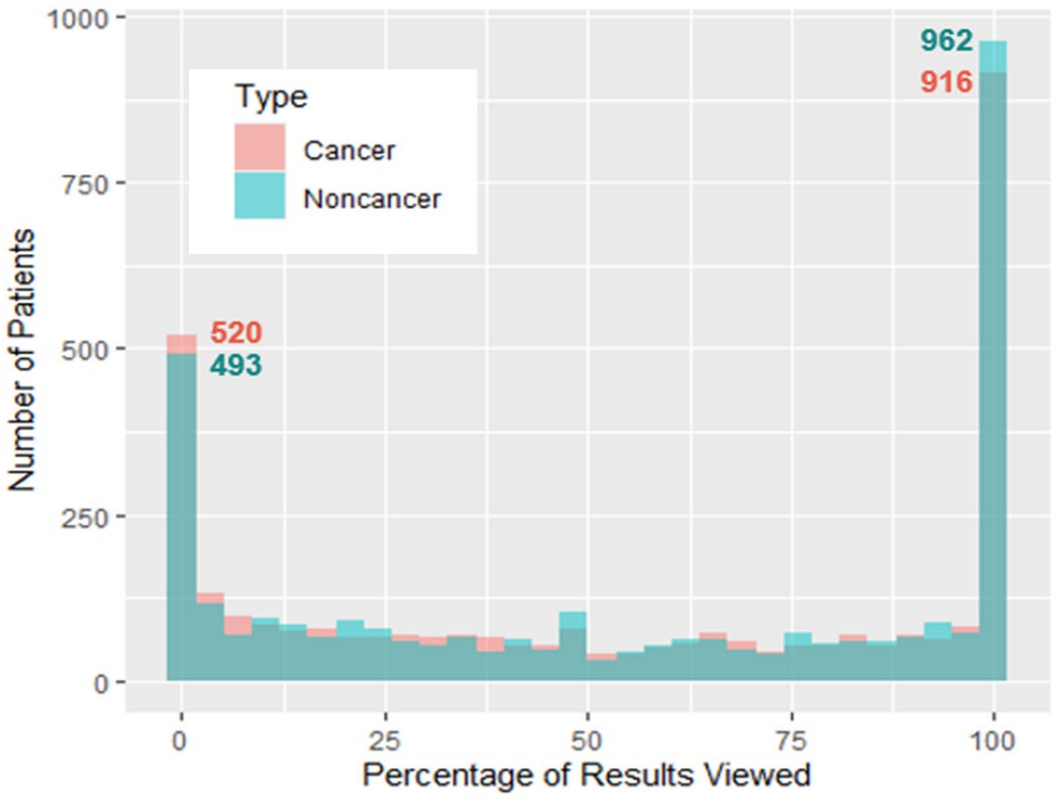

Distribution of viewing noncancer results, in teal, is skewed more to the right than viewing cancer results, in peach. For example: While 962 patients viewed almost all (97%-100%) of their noncancer results, only 916 viewed almost all of their cancer results. There were 493 patients that viewed almost none (0-3%) of noncancer results while more patients (520) viewed almost no cancer results.

B. Years-of-activity

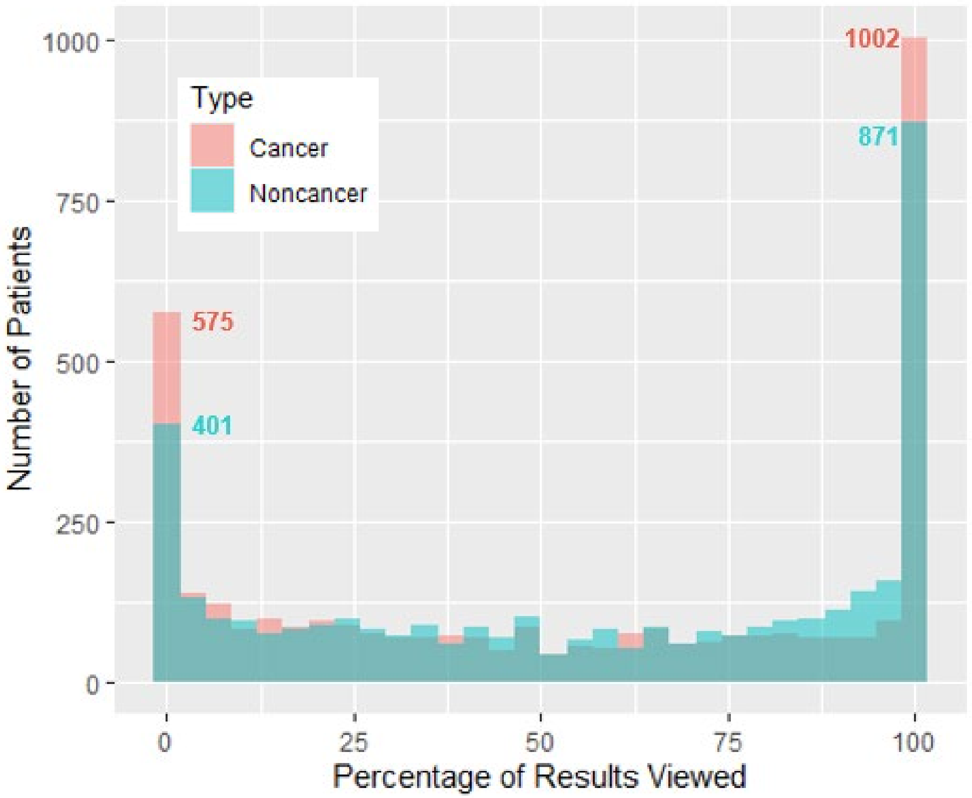

Distribution of viewing noncancer results, in teal, is generally skewed more to the right than viewing cancer results, in peach. For example: While 401 patients viewed almost none of noncancer results (0-3%), more patients (575) viewed almost no cancer results. Contrary to year-of-diagnosis (A), less patients viewed almost all (97%-100%) of noncancer results (871) compared to 1002 patients that viewed almost all cancer results. However, more patients viewed 75-97% of noncancer results compared to patients that viewed 75-97% of cancer results.
